# Effects of pre-existing orthopoxvirus-specific immunity on the performance of Modified Vaccinia virus Ankara-based influenza vaccines

**DOI:** 10.1038/s41598-018-24820-2

**Published:** 2018-04-24

**Authors:** Arwen F. Altenburg, Stella E. van Trierum, Erwin de Bruin, Dennis de Meulder, Carolien E. van de Sandt, Fiona R. M. van der Klis, Ron A. M. Fouchier, Marion P. G. Koopmans, Guus F. Rimmelzwaan, Rory D. de Vries

**Affiliations:** 1 Department of Viroscience, Postgraduate School of Molecular Medicine, Erasmus MC, Rotterdam, The Netherlands; 2 Centre for Infectious Disease Control (Cib), National Institute of Public Health and the Environment (RIVM), Bilthoven, The Netherlands; 3 Present Address: Research Center for Emerging Infections and Zoonoses (RIZ), University of Veterinary Medicine, Hannover, Germany

## Abstract

The replication-deficient orthopoxvirus modified vaccinia virus Ankara (MVA) is a promising vaccine vector against various pathogens and has an excellent safety record. However, pre-existing vector-specific immunity is frequently suggested to be a drawback of MVA-based vaccines. To address this issue, mice were vaccinated with MVA-based influenza vaccines in the presence or absence of orthopoxvirus-specific immunity. Importantly, protective efficacy of an MVA-based influenza vaccine against a homologous challenge was not impaired in the presence of orthopoxvirus-specific pre-existing immunity. Nonetheless, orthopoxvirus-specific pre-existing immunity reduced the induction of antigen-specific antibodies under specific conditions and completely prevented induction of antigen-specific T cell responses by rMVA-based vaccination. Notably, antibodies induced by vaccinia virus vaccination, both in mice and humans, were not capable of neutralizing MVA. Thus, when using rMVA-based vaccines it is important to consider the main correlate of protection induced by the vaccine, the vaccine dose and the orthopoxvirus immune status of vaccine recipients.

## Introduction

Recombinant viral vectors are under development as novel vaccine candidates that induce immunity to antigens of interest expressed from transgenes. Numerous vector-based vaccine candidates have been tested over the last decades, targeting a wide range of cancers or infectious diseases^1–5^. Modified vaccinia virus Ankara (MVA), a member of the *Orthopoxvirus* genus, is a promising vaccine vector derived from the vaccinia virus (VACV) strain chorioallantois vaccinia virus Ankara through extensive serial passaging in chicken embryo fibroblasts (CEF). This serial passaging resulted in the loss of approximately 15% of the parental genome at so-called ‘deletion sites’^6,7^, allowing for easy generation of recombinant (r)MVA by insertion of one or multiple genes encoding antigens of interest into the MVA genome. Furthermore, MVA has lost the ability to replicate in most mammalian cell types, leading to an excellent safety record in humans and even safe administration to immunocompromised subjects^8–11^. Since MVA is a replication-deficient vector, it infects cells and drives endogenous expression of antigens under the control of a VACV promotor, resulting in efficient antigen presentation and subsequent induction of antigen-specific B and T cell responses^3,4,12^.

There is considerable interest in the development of novel influenza vaccines that induce broadly protective or ‘universal’ immunity against different subtypes of influenza A viruses. Accumulation of mutations in the surface proteins of seasonal influenza viruses (antigenic drift) and the occasional zoonotic introduction of novel influenza viruses into the human population (antigenic shift) complicate the timely production of ‘classical’ influenza vaccines that antigenically match seasonal or pandemic viruses^13–17^. Furthermore, in case of a pandemic outbreak caused by a newly emerging influenza virus, novel technology is required to rapidly produce large batches of vaccines. rMVA vaccines expressing one or multiple influenza virus antigens could potentially fulfill both of these needs. Currently, rMVA-based vaccines expressing various wild-type and modified influenza virus antigens are evaluated in animal models and clinical trials and have shown promising results^3–5^.

A potential drawback for the use of orthopoxvirus-based vaccines is that a proportion of the adult human population has immunity against the vaccine vector due to smallpox vaccination campaigns that were conducted until the mid 1970s and ultimately led to the eradication of smallpox^18^. In general, orthopoxvirus-specific immunity induced by smallpox vaccination is long-lived with slowly declining T cell responses (half-life of 8–15 years) and antibody responses that are maintained up to 75 years after vaccination^19^. In addition to orthopoxvirus-specific immunity induced by the historic use of smallpox vaccines, efficient induction of immunity by rMVA-based vaccines often requires repeated administration, which induces immunity not only to the antigen of interest but also against the vaccine vector^20^. There is considerable concern for interference of orthopoxvirus-specific pre-existing immunity with subsequent rMVA-based vaccinations, resulting in reduced vaccine immunogenicity and efficacy.

Previously, pre-existing vaccine vector-specific immunity was shown to interfere with VACV-^21^, fowlpox virus-^22^ and adenovirus-based vaccines^23,24^. In contrast to MVA, these vector-based vaccines are replication-competent in their respective hosts and therefore potentially more sensitive to pre-existing vaccine vector-specific immunity. Thus far, evidence for interference of pre-existing orthopoxvirus-specific immunity with rMVA vaccination is ambiguous. Some studies in mice and macaques showed that pre-existing immunity induced by either VACV or MVA had a negative effect on the induction of antigen-specific humoral and/or cellular immune responses by rMVA-based vaccines. However, despite the observed negative effects, pre-existing orthopoxvirus-specific immunity was not considered to interfere with rMVA-based vaccination^25–28^. Furthermore, results obtained in humans are also contradictory: orthopoxvirus-specific immunity was boosted by multiple rMVA vaccinations and was shown to have a negative effect on the magnitude of the antigen-specific humoral and cellular immune response. However, in all cases individuals responded to vaccination by either initial induction or boosting of antigen-specific immunity^20,29^. This indicates that rMVA-based vaccines remain immunogenic, even in the presence of vector-specific pre-existing immunity. Thus, despite the fact that claims of potential interference by pre-existing vector immunity on immunogenicity of rMVA-based vaccines are made in the literature, the topic has not been addressed sufficiently and studies have led to contradictory results.

In this study, we addressed the effect of pre-existing immunity to MVA, VACV or influenza virus on the performance of rMVA-based influenza vaccines by evaluating induction of immune responses and the protective capacity from a lethal challenge with an influenza virus. Mice were primed with either wild-type (wt)MVA – to mimic a scenario of multiple exposures to MVA, for example in a repeated vaccination regimen – or VACV, representing people who have been vaccinated against smallpox. Furthermore, (cross-)neutralizing activity of MVA- or VACV-specific antibodies against rMVA-based vaccines was assessed using mouse and human sera. Importantly, the protective capacity of an rMVA vaccine expressing a hemagglutinin (HA) gene homologous to the H5N1 challenge virus was not hampered by the presence of pre-existing immunity to MVA, VACV or influenza virus. However, pre-existing orthopoxvirus-specific immunity interfered with induction of antigen-specific antibody responses under specific conditions and had a detrimental effect on the induction of antigen-specific T cell responses.

## Results

### VACV and H1N1pdm09 virus dose-finding

Sub-lethal doses of VACV and pandemic influenza virus (H1N1pdm09) were determined in dose-finding experiments in C57BL/6 mice. Inoculation of mice with 10^4^–107 plaque forming units (PFU) VACV-Elstree by tail scarification led to weight loss (Fig. S1A), concurrent with the appearance of blisters at the site of inoculation in all mice (Fig. S1B). Similar levels of VACV-specific antibody responses were detected in all groups two weeks after inoculation (Fig. S1c). In addition, VACV- and MVA-specific CD4^+^ and CD8^+^ T cell responses were detected with a trend of stronger T cell responses at increasing infectious doses (Fig. S1D,E). A dose of 10^7^ PFU VACV was considered the optimal sub-lethal priming dose for subsequent experiments.

In contrast to VACV, intranasal (IN) inoculation of mice with incrementing doses of H1N1pdm09 virus resulted in severe weight loss (Fig. S2A). Mortality was observed in mice inoculated with 10^5^ and 10^6^ tissue-culture infectious dose −50 (TCID_50_) of H1N1pdm09 virus (Fig. S2B). Optimal induction of hemagglutination inhibition (HI) antibody responses (Fig. S2C) and T cell responses (Fig. S2D) without mortality was observed after inoculation with 10^4^ TCID_50_, which was therefore considered the optimal dose for subsequent sub-lethal priming infections.

### Induction of pre-existing orthopoxvirus-specific and influenza virus-specific immunity

**Figure 1:**
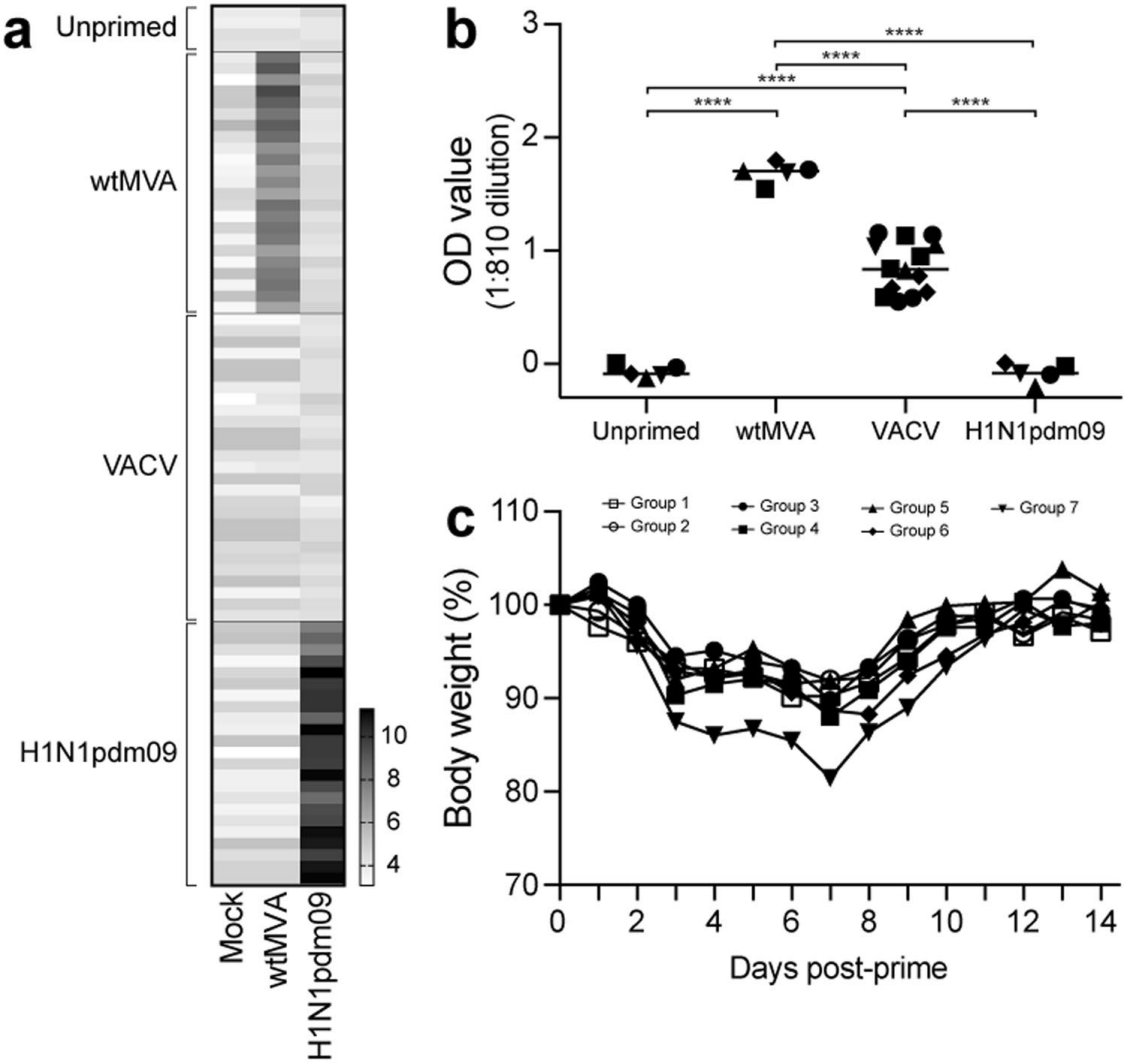
Induction of orthopoxvirus-specific or influenza virus-specific immunity by priming. (**A**) Sera from individual mice obtained 4 weeks after the last priming (week 8) were assessed by PA for presence of wtMVA- or H1N1pdm09-specific antibodies. MVA-specific antibodies were detected with a wtMVA-infected cell lysate, mock-infected baby hamster kidney (BHK)-21 cell lysate was included as negative control. Each horizontal line represents an individual animal. Antigens (x-axis) and priming groups (y-axis) are indicated. Scale shows 2-log transformed titers. (**B**) VACV-specific serum antibody responses were determined by ELISA using VACV-infected HeLa cell lysate. The background signal on mock-infected cell lysate was subtracted. Individual sera from VACV-primed mice were used where possible. Serum from unprimed, wtMVA-primed or H1N1pdm09-primed mice was pooled due to limited serum availability. Mean per priming group is indicated. Statistically significant differences were determined using a one-way ANOVA with multiple comparisons. ****p < 0.0001. (**C**) Mean body weight per group (n = 6, group 7 n = 5) after IN inoculation with 10^4^ TCID_50_ H1N1pdm09. No statistically significant differences between the groups were detected with a repeated measures ANOVA model.

According to the indicated priming regimens (Table 1, week 0 and/or 4) orthopoxvirus-specific or H1N1pdm09 influenza virus-specific immunity was induced. Four weeks after the last priming inoculation (week 8), induction of orthopoxvirus- or influenza virus-specific immunity was assessed by measuring serum antibody responses by protein array (PA) and ELISA. Priming with wtMVA or H1N1pdm09 influenza virus induced homologous antibody responses measured by PA (Fig. 1A). Serum antibodies reactive with wtMVA could not be detected in VACV-primed mice in this assay. Therefore, induction of VACV-specific antibodies by VACV priming was confirmed by ELISA (Fig. 1B). Notably, orthopoxvirus- or influenza virus-specific antibody responses were not detected in unprimed mice (Fig. 1A,B). In addition to detection of serum antibodies, H1N1pdm09 virus infection was confirmed by monitoring body weight of mice two weeks post-priming (Table 1, subgroup d). H1N1pdm09-virus inoculated mice lost body weight up to 7 days post-inoculation (dpi) and had regained their original weight at 11 dpi (Fig. 1C). In summary, priming with wtMVA, VACV or H1N1pdm09 was successful and induced detectable pre-existing immunity against the respective viruses in C57BL/6 mice.

**Table 1:** Experimental design.

Group	Week 0	Week 4	Week 8	Week 12	Week 16
	Prime 1	Prime 2	Vaccination 1	Vaccination 2	Challenge
1	a) —	a) —	rMVA-NP	rMVA-NP	X
	b) wtMVA	b) wtMVA			
	c) —	c) VACV			
	d) —	d) H1N1pdm09			
2	a) —	a) —	rMVA-H5	rMVA-H5	X
	b) wtMVA	b) wtMVA			
	c) —	c) VACV			
	d) —	d) H1N1pdm09			
3	a) —	a) —	rMVA-H1	rMVA-H1	H5N1
	b) wtMVA	b) wtMVA			
	c) —	c) VACV			
	d) —	d) H1N1pdm09			
4	a) —	a) —	rMVA-H3	rMVA-H3	H5N1
	b) wtMVA	b) wtMVA			
	c) —	c) VACV			
	d) —	d) H1N1pdm09			
5	a) —	a) —	PBS	rMVA-H5	H5N1
	b) wtMVA	b) wtMVA			
	c) —	c) VACV			
	d) —	d) H1N1pdm09			
6	a) —	a) —	rMVA-H5*	rMVA-H5	H5N1
	b) wtMVA	b) wtMVA			
	c) —	c) VACV			
	d) —	d) H1N1pdm09			
7	a) —	a) —	rMVA-H1	rMVA-H5	H5N1
	b) wtMVA	b) wtMVA			
	c) —	c) VACV			
	d) —	d) H1N1pdm09			

C57BL/6 mice (n = 6 per subgroup) were unprimed or primed with 10^8^ PFU wtMVA (two primings, subgroups b), 10^7^ PFU VACV (one priming, subgroups c) or 10^4^ TCID_50_ H1N1pdm09 (one priming, subgroups d). At week 8 and 12, mice were vaccinated with 10^8^ PFU of the indicated rMVA-based vaccine expressing influenza virus nucleoprotein (NP) or HA. Groups 1 and 2 were euthanized at week 13 and 14, respectively, to assess the effect of priming on induction of antigen-specific T cells by vaccination. The remainder of the mice was challenged with a lethal dose of 10^3^ TCID_50_ A/Vietnam/1194/04 (H5N1) influenza virus at week 16. *Mice were vaccinated with 10^7^ PFU rMVA-H5.

### Pre-existing orthopoxvirus-specific immunity had limited effect on induction of antigen-specific antibody responses by rMVA

**Figure 2:**
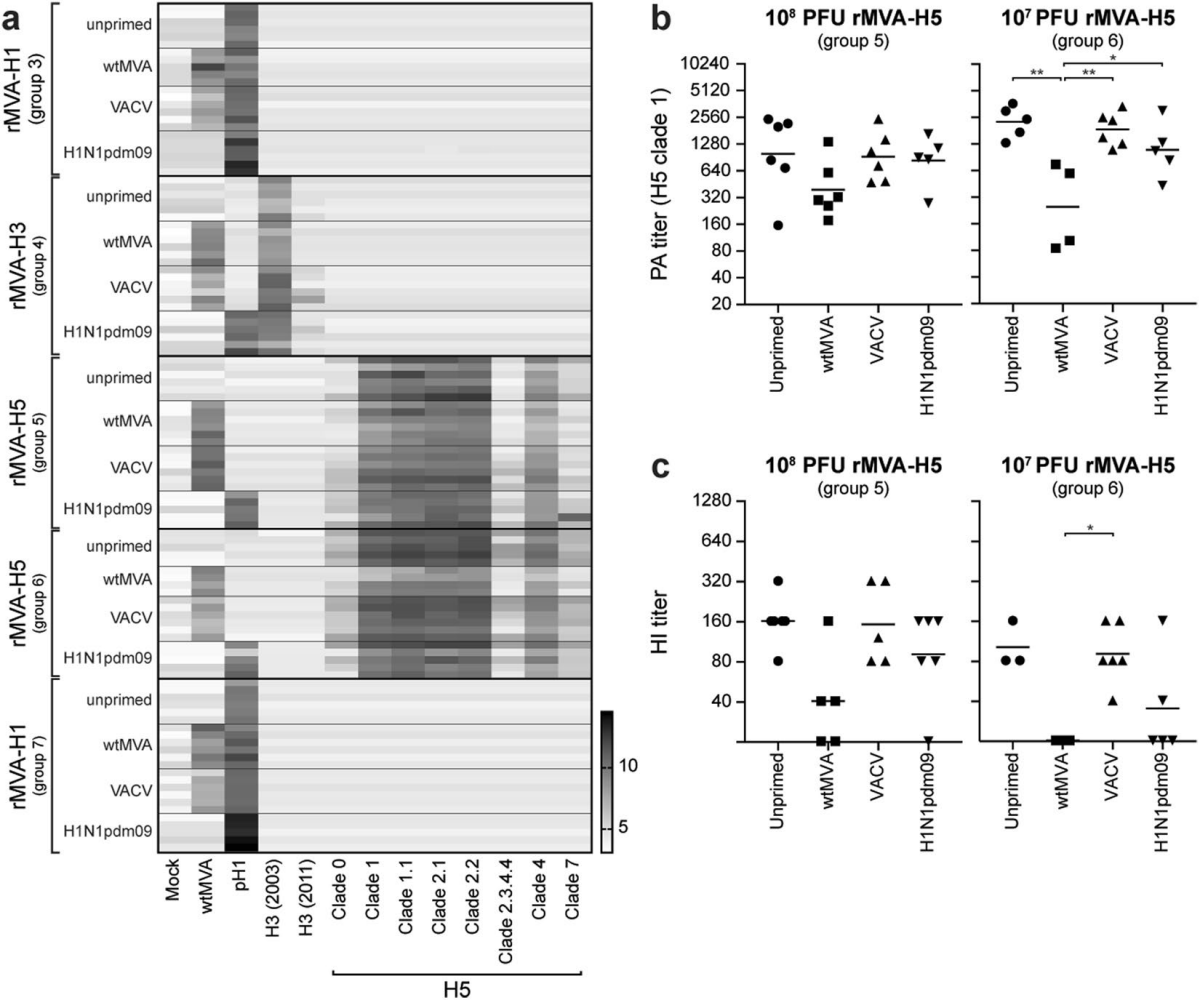
Effect of pre-existing immunity on induction of serum antibody responses by a single rMVA vaccination. (**A**–**B**) Serum antibody responses against wtMVA and HA1 from H1N1pdm09, 2003 H3N2, 2011 H3N2 or H5 influenza viruses from the indicated clades were determined using PA 4 weeks after the first vaccination (group 3, 4, 6 & 7 week 12, group 5 week 16). Mock-infected BHK-21 cell lysates were used as negative control for the wtMVA-infected cell lysates. Each horizontal line represents an individual animal grouped according to the vaccination group (rMVA-H1, rMVA-H3 or rMVA-H5) and priming subgroup (unprimed, wtMVA-primed, VACV-primed or H1N1pdm09-primed) indicated on the y-axis. Each vertical bar represents a different antigen (indicated on the x-axis). Scale represents 2-log titers as determined by PA, darker colors indicate higher antibody titers. (**B**) PA titers against H5 from A/Vietnam/1194/04 (clade 1) four weeks after rMVA-H5 vaccination with a high dose (10^8^ PFU, group 5, week 16) or a low dose (10^7^ PFU, group 6, week 12). Mean per priming group is indicated. Statistically significant differences were determined using a one-way ANOVA with multiple comparisons. *p = 0.0257, **p < 0.0017. (**C**) HI titers against influenza virus A/Vietnam/1194/04 were determined for each individual animal four weeks after rMVA-H5 vaccination with a high dose (10^8^ PFU, group 5, week 16) or a low dose (10^7^ PFU, group 6, week 12). Mean is indicated. Statistically significant differences were determined using a Kruskal-Wallis test. *p = 0.0401.

To determine the effect of pre-existing immunity on rMVA vaccine immunogenicity, unprimed and primed mice were vaccinated with rMVA expressing influenza virus nucleoprotein (NP) or HA (Table 1). Serum antibody responses against wtMVA and various HA1 subunits (HA from H1N1pdm09, H3N2 isolate from 2003 and 2011, and a selection of H5Nx viruses) after a single rMVA vaccination were determined by PA. As expected, rMVA vaccination consistently boosted the MVA-specific antibody response in mice primed with wtMVA or VACV (Fig. 2A, compare wtMVA response of wtMVA-/VACV-primed mice with unprimed/H1N1pdm09 primed mice in all groups). Furthermore, boosting of H1N1pdm09-specific antibodies was observed in mice primed with H1N1pdm09 virus and subsequently vaccinated with rMVA-H1 (Fig. 2A, group 3 and 7).

Induction of serum antibody responses against the corresponding antigen (H1pdm09 for rMVA-H1 and H3 [2003] for rMVA-H3 vaccination), was not hampered by either orthopoxvirus- or influenza virus-specific pre-existing immunity (Fig. 2A). Antibodies against the heterologous H3 (2011) were not detected after a single vaccination with rMVA-H3. In contrast, a single rMVA-H5 vaccination led to induction of antibody responses to the homologous H5 antigen, as well as heterologous H5 antigens. In wtMVA-primed mice, lower antibody titers against the HA1 of all tested H5 clades, including the homologous A/Vietnam/1194/04 (H5N1, clade 1), was observed compared to unprimed, VACV- or H1N1pdm09-virus primed mice. This effect was particularly detected when a lower dose of rMVA-H5 (10^7^ PFU, Table 1, group 6 at week 12) was used for the initial vaccination and to a lesser extent with the use of a higher vaccine dose (10^8^ PFU, Table 1, group 5 at week 16) (Fig. 2A,B). These results were confirmed by HI assay, which is a good proxy for influenza virus neutralization. Corresponding to the PA data, MVA-specific pre-existing immunity negatively affected the HI antibody response to influenza virus A/Vietnam/1194/04 after a single rMVA-H5 vaccination, especially when a low dose was used (Fig. 2C).

**Figure 3:**
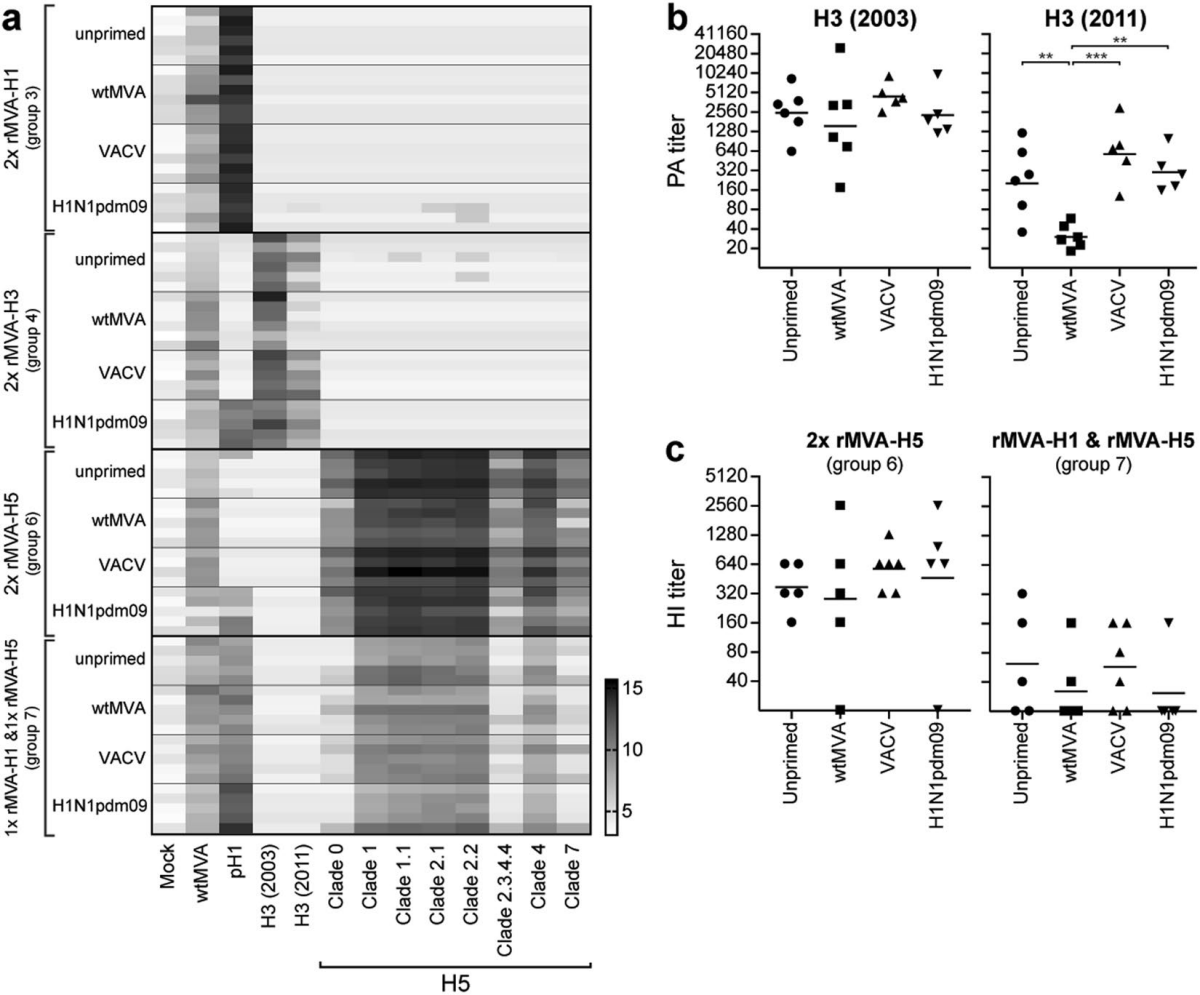
Effect of pre-existing immunity on induction of serum antibody responses after two rMVA vaccinations. (**A**–**B**) Serum antibody responses against wtMVA and HA1 from H1N1pdm09, 2003 H3N2, 2011 H3N2 or H5 influenza viruses from the indicated clades were determined using protein array 4 weeks after the second vaccination (week 16). Each horizontal line represents an individual animal grouped according to the vaccination group (2x rMVA-H1, 2x rMVA-H3, 2x rMVA-H5 or rMVA-H1 followed by rMVA-H5) and priming subgroup (unprimed, wtMVA-primed, VACV-primed or H1N1pdm09-primed) indicated on the y-axis. Each vertical bar represents a different antigen (indicated on the x-axis). Scale represents the 2-log as determined by protein array, darker colors indicate higher antibody titers. (**B**) Protein array titers for each individual animal against an antigenically similar H3 from 2003 or distinct H3 from 2011 four weeks after the second rMVA-H3 vaccination (group 4). Mean per priming group is indicated. Statistically significant differences were determined using a one-way ANOVA with multiple comparisons. **p < 0.0086, ***p = 0.0003. (**C**) HI titers against influenza virus A/Vietnam/1194/04 were determined for each individual animal four weeks after the second vaccination with rMVA. Mean per priming group is indicated.

A second vaccination with rMVA (Table 1, week 16) boosted serum antibody responses to wtMVA and influenza viruses of interest. Similar to antibody responses induced by a single vaccination, antibody responses against the corresponding antigen after two vaccinations with rMVA-H1 or rMVA-H3 were not affected by pre-existing immunity (Fig. 3A,B, group 3–4). The second vaccination with rMVA-H3 induced cross-reactive antibody responses against an antigenically distinct H3 (2011). Notably, this cross-reactive response was detected in all subgroups, but was lower in mice with MVA-specific pre-existing immunity (Fig. 3A,B). Furthermore, in contrast to the antibody response after a single rMVA-H5 vaccination, the response after two rMVA-H5 vaccinations (Table 1, group 6) or an rMVA-H1 vaccination followed by 10^8^ PFU rMVA-H5 (Table 1, group 7) detected by either PA or HI was not affected by pre-existing immunity to the vector (Fig. 3A and C). Interestingly, recurrent vaccination with rMVA expressing different antigens (rMVA-H1 and rMVA-H5) still lead to efficient induction of antibody responses against both HAs. In conclusion, an effect of pre-existing MVA-specific, but not VACV- or influenza virus-specific, immunity on induction of humoral responses by rMVA vaccination was observed under specific conditions.

### Induction of antigen-specific T cell responses by rMVA is prevented by pre-existing orthopoxvirus-specific immunity

To determine the effect of pre-existing immunity on rMVA-induced antigen-specific T cell responses, splenocytes were obtained from unprimed and primed mice one or two weeks after the second rMVA-NP or rMVA-H5 vaccination, respectively (Table 1, group 1–2). Antigen-specific CD8^+^ T cell responses were determined by measuring the number of interferon (IFN)-γ producing splenocytes after stimulation with synthetic peptide NP_366–374_, an immunodominant CD8^+^ T cell epitope. Furthermore, the H5-specific CD4^+^ T cell response was determined after stimulation of splenocytes with full-length HA protein from H5N1 influenza viruses A/Vietnam/1194/04 (clade 1) or A/Indonesia/5/05 (clade 2.1).

**Figure 4:**
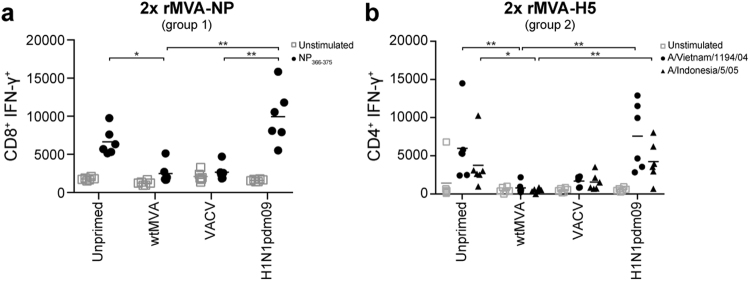
Pre-existing orthopoxvirus-specific immunity inhibits rMVA-induced antigen-specific T cell responses. (**A**) Splenocytes collected 1 week after the second rMVA-NP vaccination were unstimulated or stimulated with NP_366–374_ synthetic peptide. The number of IFN-γ producing CD3^+^CD8^+^ splenocytes was measured. (**B**) Splenocytes collected two weeks after the second rMVA-H5 vaccination were unstimulated or stimulated with purified HA protein from H5N1 influenza virus A/Vietnam/1194/04 or A/Indonesia/5/05. The number of IFN-γ producing CD3^+^CD4^+^ splenocytes was measured. Mean of each priming group is indicated. Statically significant differences per simulant were determined using a Kruskal-Wallis test. *p < 0.0478, **p < 0.0076.

rMVA-NP vaccination efficiently induced antigen-specific CD8^+^ T cell responses in unprimed and H1N1pdm09-primed mice but failed to induce NP-specific CD8^+^ T cells in mice with orthopoxvirus-specific pre-existing immunity, induced by either wtMVA or VACV priming (Fig. 4A). Similar observations were made in animals vaccinated with rMVA-H5: unprimed and H1N1pdm09 primed animals developed HA-specific CD4^+^ T cell responses against both the homologous (A/Vietnam/1194/04) and heterologous HA (A/Indonesia/5/05), but pre-existing orthopoxvirus-specific immunity had a detrimental effect on the induction of H5-specific CD4^+^ T cell responses (Fig. 4B).

### Pre-existing orthopoxvirus-specific immunity impaired the protective efficacy of rMVA-based vaccines against challenge with a heterologous but not homologous virus

**Figure 5:**
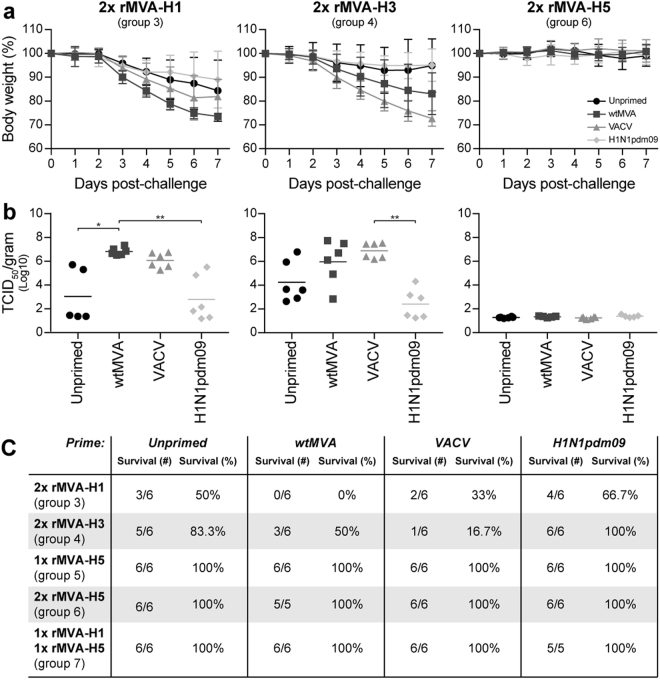
Pre-existing immunity does not impair protective capacity of rMVA-H5 vaccination. Four weeks after the last rMVA vaccination, mice were challenged with a lethal dose H5N1 influenza virus. (**A**) Body weight over time for each of the priming groups for group 3 (two rMVA-H1 vaccinations), group 4 (two rMVA-H3 vaccinations) and group 6 (two rMVA-H5 vaccinations). Mean and standard deviation (SD) per priming group are shown. (**B**) Viral load in the lungs shown as TCID_50_ per gram lung for each individual animal for group 3, group 4 and group 6. Mean of each priming group is shown. Statistically significant differences were determined using a Kruskal-Wallis test. *p = 0.0152, **p < 0.0065. (**C**) Survival after lethal H5N1 influenza virus challenge. Mice were euthanized at >25% body weight loss. The absolute number (#; live mice/total mice) and the percentage (%) of live animals at seven days post-challenge have been indicated.

Four weeks after the final vaccination, rMVA-H1, rMVA-H3 and rMVA-H5 vaccinated mice were challenged with a lethal dose of influenza virus H5N1 (A/Vietnam/1194/04) in order to determine the effect of pre-existing immunity on the protective capacity of rMVA-based influenza vaccines (Table 1, group 3–7). As expected, rMVA-H1 and rMVA-H3 vaccination did not fully protect against an H5N1 influenza virus challenge, which was reflected by loss of body weight, lower survival rates and high viral loads in the lungs (Fig. 5). However, a limited level of cross-protection was observed after rMVA-H1 or rMVA-H3 vaccination in unprimed and H1N1pdm09-primed animals, which was not observed in mice primed with VACV or wtMVA (Fig. 5). Notably, pre-existing orthopoxvirus-specific or influenza virus-specific immunity did not interfere with the protective capacity of rMVA vaccines expressing the homologous HA gene of the H5N1 challenge virus since all mice that received at least one rMVA-H5 vaccination were fully protected from lethal H5N1 challenge (Figs 5 and S3**)**.

### Pre-existing MVA-specific, but not VACV-specific, antibodies have MVA-neutralizing capacities

**Figure 6:**
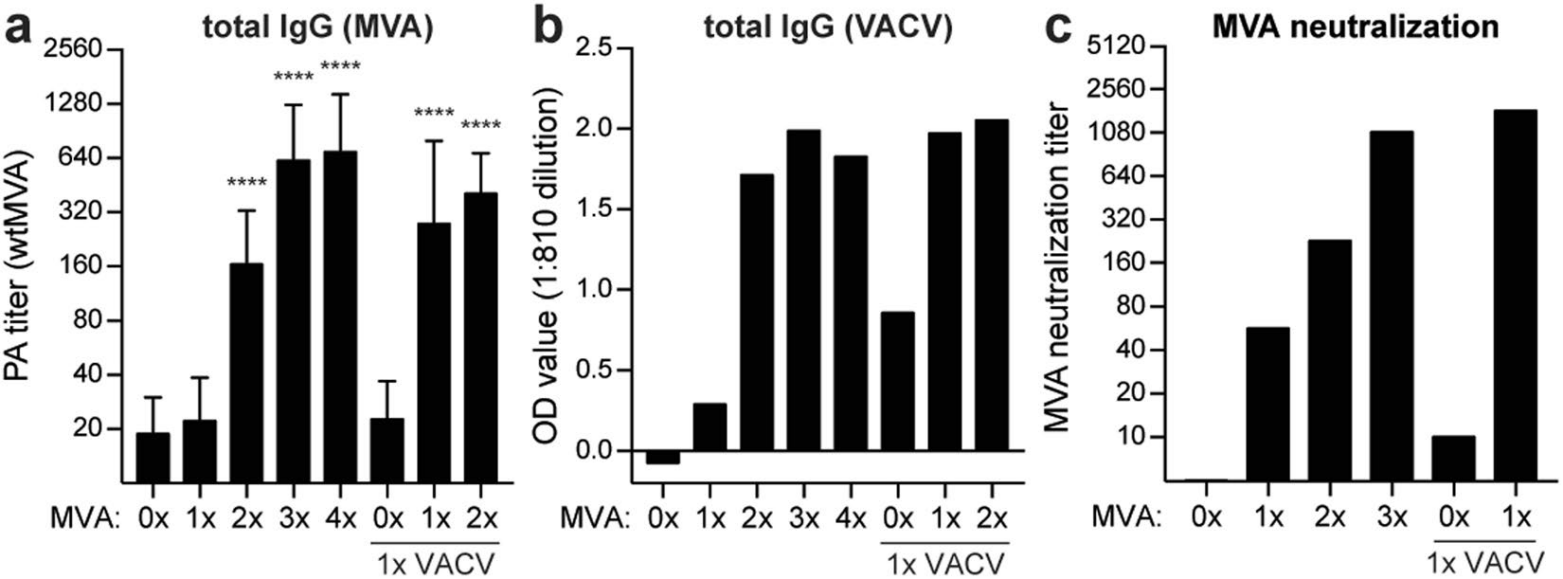
MVA-specific antibodies showed neutralizing capacity. MVA and VACV-specific antibody responses were determined in sera obtained four weeks post-priming (week 8) or post-vaccination (week 12&16). (**A**) MVA-specific serum antibody responses were determined using wtMVA-infected BHK-21 cell lysates on PA. The mean and SD are indicated. Statistical differences were determined relative to the ‘0 × MVA’ sample using a Kruskal-Wallis test. ****p < 0.0001. (**B**) Serum antibody responses against VACV were measured by ELISA using VACV-infected HeLa cell lysate. The background signal on mock-infected HeLa cell lysates was subtracted. Due to limited serum availability serum was pooled (n = 3–6) per subgroup. The mean of n = 2–17 pools is shown, except for ‘3 × MVA’ which shows data from a single pool. (**C**) Serum antibody responses in group 5 (one vaccination with rMVA-H5) and group 6 (two vaccinations with rMVA-H5) were examined for wtMVA neutralizing capacity by a plaque reduction assay on CEF. Due to limited serum availability serum was pooled (n = 2–5) per subgroup. The mean of n = 2 pools is shown, except for ‘3 × MVA’ and ‘1 × VACV, 0 × MVA’ which show data from a single pool.

Whether antibodies induced by single or multiple MVA or VACV exposures have the capacity to recognize and neutralize MVA was investigated *in vitro* by ELISA and a virus neutralization assay. Mice that received a single exposure to either MVA or VACV did not induce any detectable MVA-specific serum antibody responses. In contrast, in mice that were exposed to MVA at least twice or once to VACV followed by at least one rMVA exposure, MVA-specific antibodies were detected. Notably, the MVA-specific antibody response was not boosted in response to additional exposures after the third MVA exposure (Fig. 6A). Similar observations were made in a VACV-specific ELISA, where at least two vaccinations with MVA or once with VACV followed by MVA led to detectable VACV-reactive antibodies (Fig. 6B). A booster effect of rMVA-based vaccinations after VACV priming was observed in both MVA- and VACV-specific antibody responses (Fig. 6A,B). Only sera obtained from mice exposed to MVA, but not from mice exposed exclusively to VACV, were capable of neutralizing MVA *in vitro* (Fig. 6C).

### Sera from humans shortly following MVA vaccination, but not 40 years after smallpox vaccination, are capable of neutralizing MVA

**Figure 7:**
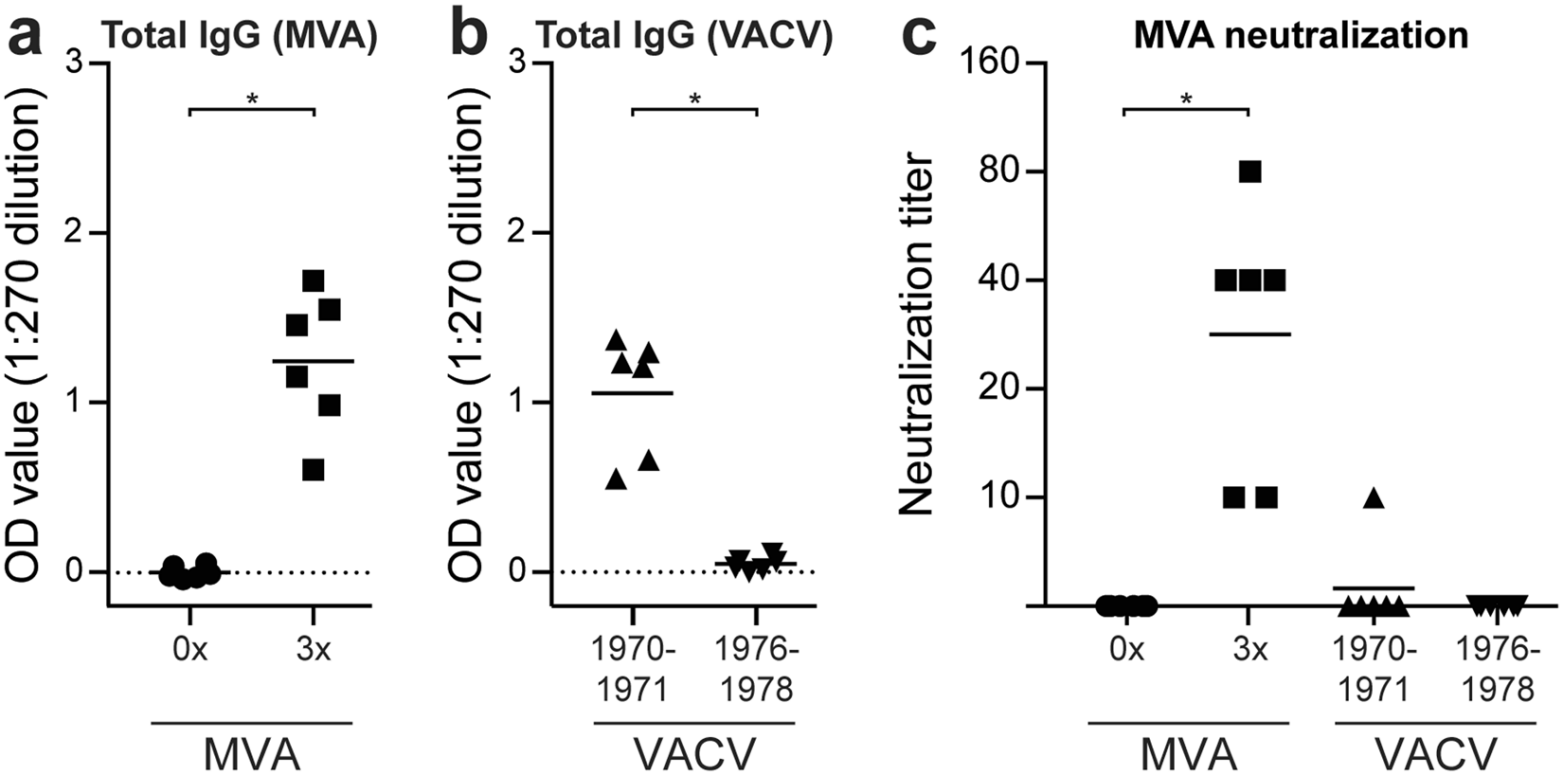
Human rMVA-based, but not VACV, induced MVA-specific neutralizing antibody responses. (**A**,**B**) Serum antibody responses against MVA (A) and VACV (**B**) were measured by ELISA using infected BHK-21 or HeLa cell lysates, respectively. The background of the respective mock-infected cell lysate was subtracted. (**C**) Neutralizing capacity of orthopoxvirus-specific antibodies was determined by a plaque reduction assay on CEF using rMVA-GFP. The data shown are representative of three independent experiments. The mean per group is indicated. Statistical significant differences were determined using a Wilcoxon matched-pairs signed rank test (rMVA vaccination samples) or Mann-Whitney test (VACV vaccination samples). *p < 0.0312. **p = 0.0022.

Next, the presence of orthopoxvirus-specific antibodies and their neutralizing capacity was assessed using serum obtained from humans vaccinated with either VACV ( ± 40 years post-vaccination) or rMVA (4 weeks after the third vaccination^20^). MVA-specific antibody responses were detected four weeks after vaccination in individuals that received multiple vaccinations with 10^8^ PFU rMVA-H5^20^ (Fig. 7A). Furthermore, sera from VACV-vaccinated individuals (born between 1970–1971) and unvaccinated controls (born between 1976–1978) were probed for the presence of VACV-specific antibodies^30^. Almost four decades after vaccination, VACV-specific antibodies were still detected in VACV-vaccinated individuals, but not in the controls that were born 2–4 years after the smallpox vaccination campaign was terminated (Fig. 7B). Notably, sera obtained from rMVA vaccinated donors neutralized MVA efficiently *in vitro*, whereas sera obtained from VACV-vaccinated individuals were not capable of neutralizing an MVA infection (Fig. 7C). Although this is of course potentially due to waning of VACV-specific antibodies and does not necessarily reflect absence of cross-reactivity, this reflects the physiological situation at this time.

## Discussion

Although it has been frequently suggested that pre-existing immunity to orthopoxviruses can interfere with the immunogenicity and efficacy of rMVA-based vaccines, the issue has not been addressed sufficiently in a well-controlled fashion. In this study, we investigated the performance of rMVA-based influenza vaccines in the presence or absence of pre-existing immunity to orthopoxviruses or influenza virus in mice and evaluated orthopoxvirus-specific immune responses after MVA or VACV vaccination in humans.

Induction of orthopoxvirus-specific antibodies upon priming mice with wtMVA or VACV was confirmed by PA and ELISA using wtMVA- or VACV-infected cell-lysates as antigens. Interestingly, VACV-specific antibodies did not cross-react with MVA in our PA assay, whereas MVA-specific antibodies clearly cross-reacted with VACV, as demonstrated by ELISA. These results are in accordance with a previous study^31^ and similar results were obtained with human sera, where VACV-specific antibody responses cross-reacted with MVA only to a limited extent. In our study, the differential response can be explained by the fact that wtMVA-primed mice received a booster immunization inducing a MVA-specific recall response opposed to induction of a primary responses in the VACV-primed mice. Indeed, we observed that a single vaccination with rMVA expressing H1, H3 or H5 in unprimed mice did not induce detectable levels of MVA-specific antibodies, whereas rMVA vaccination of VACV-primed mice led to a boosting of MVA-specific antibodies. This corresponds to data obtained in humans, which show that MVA-specific antibodies were boosted upon MVA vaccination of either smallpox or rMVA vaccinated individuals^20,31^.

Pre-existing orthopoxvirus-specific immunity in mice affected humoral immune responses induced by rMVA influenza vaccines to a limited extent. Effects were exclusively observed in wtMVA-primed mice and only under ‘suboptimal’ conditions. When a lower rMVA-H5 vaccine dose was used, wtMVA-primed mice had reduced antibody titers to all tested H5 clades compared to the other priming groups. The reduced H5-specific antibody response observed in mice with MVA-specific pre-existing immunity was overcome by a second immunization with 10^8^ PFU of rMVA-H5, as was previously reported^27^. Furthermore, in wtMVA-primed mice the response to an antigenically distinct H3N2 virus was significantly lower after two rMVA-H3 vaccinations compared to unprimed animals. However, pre-existing immunity had no effect on the magnitude of the antibody response to a corresponding H3 antigen induced by one or two rMVA-H3 vaccinations. Importantly, negative effects on the induction of antigen-specific antibody responses induced by rMVA were exclusively observed after wtMVA priming. VACV-specific pre-existing immunity never had any effect on the antigen-specific antibody response.

In addition to antibody responses, the induction of antigen-specific T cell responses after rMVA vaccination in the absence or presence of pre-existing immunity was assessed. Virus-specific T cells contribute significantly to protective immunity against virus infections and can reduce duration and severity of disease^32–34^. Induction of T cells to influenza virus by vaccines is particularly attractive, since these mainly recognize epitopes in conserved internal proteins and can therefore afford cross-protection against various influenza viruses of different subtypes (heterosubtypic immunity)^35–38^. Our results indicated that induction of influenza virus-specific T cell responses was severely hampered by presence of MVA- or VACV-specific pre-existing immunity in mice. These findings are in concordance with previous studies that examined the immunogenicity of rMVA expressing human immunodeficiency virus (HIV) antigens in both mice^39^ and macaques^28,40^ in the presence of pre-existing vector-specific immunity. In contrast, a recent clinical trial reported efficient induction of cytomegalovirus (CMV)-specific T cell responses with rMVA in VACV-vaccinated individuals. The authors claimed that pre-existing VACV-specific immunity did not affect immunogenicity of rMVA, however, only a limited number of study subjects with pre-existing immunity was studied and their orthopoxvirus immune status was solely defined by date of birth and not confirmed with immunological assays^41^. Furthermore, rMVA expressing NP and matrix 1 (M1) genes from influenza virus has been shown to induce T cell responses in humans^42,43^, even in the elderly that potentially have orthopoxvirus-specific immunity^44^. Although this suggests that T cell responses can be induced in humans with VACV-specific immunity, the immune status of study subjects was not verified in these studies and appropriate control groups were lacking.

Pre-existing orthopoxvirus- or influenza virus-specific immunity did not affect survival of rMVA-H5 vaccinated mice after a lethal H5N1 influenza virus challenge. Protection was most likely mediated by antigen-specific neutralizing antibodies, which have been shown to be the main correlate of protection induced by this rMVA-H5 vaccine^20,45–48^, and were unaffected by the presence of pre-existing immunity at week 16. In contrast, mice that were vaccinated with either rMVA-H1 or rMVA-H3 were partially protected from lethal H5N1 influenza virus challenge. A limited level of protection against H5N1 influenza virus infection was observed in unprimed or H1N1pdm09-primed mice, most likely mediated by cross-reactive antibody or T cell responses against influenza virus induced by priming and/or vaccination. Notably, mice with pre-existing orthopoxvirus-specific immunity had higher viral loads in the lungs and more severe weight loss compared to unprimed or H1N1pdm09 primed mice. In accordance with the described immunogenicity results, we hypothesize that orthopoxvirus-specific pre-existing immunity prevented the induction of antibody and/or T cell responses by rMVA-H1 or rMVA-H3 vaccination that are cross-reactive with H5.

It has been shown previously that VACV- or MVA-based vaccination efficiently induces both orthopoxvirus-specific antibodies and T cell responses^19,20,29,49–52^ (reviewed in^53^). Hypothetically, vector-specific antibodies induced by previous immunizations could capture and neutralize rMVA virus particles upon (re-)vaccination, but non-neutralizing antibodies or orthopoxvirus-specific T cells could also play a role in interference. Interestingly, VACV-induced pre-existing immunity only interfered with induction of antigen-specific T cell responses but not antibody responses whereas MVA-induced pre-existing immunity could interfere with both. Since we have demonstrated that VACV-specific antibodies cannot cross-neutralize MVA *in vitro*, we hypothesize that interference of MVA-induced pre-existing immunity with induction of antigen-specific antibody responses is mediated by vector-specific antibodies. Similar results were obtained in humans, where VACV-induced antibodies could not neutralize MVA *in vitro* and only MVA-induced antibodies had MVA-neutralizing capacity. Follow-up adoptive transfers studies should be performed to identify the exact mechanism of interference of orthopoxvirus-specific immune responses with performance of MVA-based vaccinations.

It is important to note that our study in mice reflects a “worst-case scenario”, since a time interval of only four weeks between induction of pre-existing immunity and initial vaccination with rMVA was maintained, not allowing for waning of orthopoxvirus-specific immunity. This does not accurately reflect the human situation, where smallpox vaccination was discontinued in the mid 1970s^18^. Even though VACV-specific antibody responses were still detected in the serum of vaccinated individuals by ELISA, these antibodies did not have MVA-neutralizing capacity *in vitro*. However, the timing used in this study does reflect the ‘standard’ interval used in rMVA vaccination regimens with multiple vaccinations, in which four-week intervals are frequently observed^11,20,41^. Our results show that repeated rMVA vaccination of humans does induce MVA-specific antibodies, which have neutralizing capacities *in vitro* and therefore may interfere with the immunogenicity of subsequent vaccinations.

In conclusion, the present study aids our understanding regarding immunogenicity of MVA-based vaccines in the presence of orthopoxvirus-specific immunity. Importantly, in mice rMVA is still immunogenic in the presence of orthopoxvirus-specific immunity, however, certain scenarios exist where pre-existing immunity can interfere with vaccine efficacy. This study represents an initial foundation to evaluate the effect of pre-existing immunity in a well-controlled fashion, future studies are warranted to elucidate the mechanisms underlying this interference. When using MVA-based vaccines, it is essential to consider the orthopoxvirus immune status of vaccine recipients, the interval between vaccinations in case of repeated rMVA-based vaccination, the vaccine dose used and the main correlate of protection induced by rMVA-based vaccines to ensure induction of an optimal immune response.

## Materials and Methods

### Ethics statement

Animal experiments were conducted in strict compliance with European guidelines (EU directive on animal testing 2010/63/EU). The animal protocol was approved by an independent animal experimentation ethical review committee (Erasmus MC permit number EUR3277–02). Animal welfare was observed on a daily basis, and all invasive animal handling was performed under anaesthesia using 4% isoflurane in oxygen to minimize animal suffering. Human sera pre- and post-rMVA vaccination (three vaccinations at week 0 and 56 with 10^8^ PFU rMVA-H5, n = 6) were obtained during a randomized, double-blind phase 1/2a study at the Erasmus MC, Rotterdam, the Netherlands. The study involved adult volunteers (male/female, between ages 18–28) who provided informed consent. The study design was reviewed and approved by the Central Committee on Research involving Human Subjects in the Netherlands^20^. Furthermore, serum samples from VACV vaccinated (n = 6) and unvaccinated (n = 6) healthy individuals (male/female) were collected during a cross-sectional population-based study performed in the Netherlands from February 2006 until June 2007 (PIENTER2 study)^30^. Smallpox vaccination campaigns lasted until September 1974 in the Netherlands. To limit the inevitable age bias, sera from individuals born between 1970–1971 and 1976–1978 were selected for the VACV vaccinated and unvaccinated group, respectively. The work described here has been carried out in accordance with the code of ethics of the world medical association (declaration of Helsinki).

### Cell lines

Madin-Darby Canine Kidney (MDCK) cells were cultured in Eagle’s Minimum Essential Medium (EMEM, Sartorius Stedim BioWhittaker) supplemented with 10% fetal bovine serum (FBS, Greiner Bio-One), 20 mM HEPES (Lonza BioWhittaker), 0.1% CHNaO_3_ (Lonza BioWhittaker), and 100 μg/ml penicillin, 100U/ml streptomycin and 2mM L-Glutamine (P/S/G, Lonza). CEF were isolated from 11-day-old chicken embryos (Drost Loosdrecht BV) and passaged once before use as described previously^45^. CEF were cultured in Virus Production-Serum Free Medium (VP-SFM, Gibco) containing P/S. HeLa cells were cultured in Dulbecco’s Modified Eagle Medium (DMEM, Lonza) supplemented with 10% FBS, 20 mM HEPES, 0.1% CHNaO_3_ and P/S/G. Baby Hamster Kidney (BHK)-21 cells were cultured in DMEM supplemented with 10% FBS, 20 mM HEPES, 0.1% CHNaO_3_, 0.1 mM non-essential amino acids (NEAA, Lonza) and P/S/G. HeLa cells were cultured in DMEM supplemented with 10% FBS, 20 mM HEPES and 0.1% CHNaO_3_ and P/S/G. All cells were cultured at 37 °C in a humidified atmosphere with 5% CO_2_.

### Viruses

rMVA expressing the NP gene of influenza virus A/Puerto Rico/8/34 (PR8, rMVA-NP), the HA gene of A/Vietnam/1194/04 (rMVA-H5) or A/Netherlands/213/03 (rMVA-H3) under the control of the psynII promotor, rMVA expressing the HA gene of influenza virus A/California/4/2009 (rMVA-H1) under control of the PH5 promotor and rMVA expressing GFP under control of the P11 promotor were prepared as described previously^45,54–56^. To generate final vaccine preparations of MVA-F6 (empty vector, wtMVA) or rMVA, the viruses were propagated in CEF, purified by ultracentrifugation through 36% sucrose and resuspended in 120 mM NaCl 10 mM Tris-HCl pH 7.4. rMVA constructs were validated by PCR analysis, sequencing, plaque titration, western blot and/or flow cytometry. The VACV strain Elstree was grown in HeLa cells as described above for the rMVA constructs on CEF cells. Influenza A viruses A/Netherlands/602/09 (H1N1pdm09) and A/Vietnam/1194/04 (H5N1) were propagated and titrated (TCID_50_) in MDCK cells as described previously^57^.

### Mice

Specified pathogen free (SPF) female C57BL/6 mice 6–8 weeks of age (Charles River) were housed at biosafety level (BSL-)2 in individual ventilated cage (IVC) units during priming and vaccination (week 0–15). During the H5N1 influenza virus challenge, mice were housed in filter-top cages in negatively pressured BSL-3 isolators (week 16–17). At all times, mice had access to food and water *ad libitum*.

### H1N1pdm09 virus and VACV-Elstree dose-finding

Four groups of mice (10–12 weeks old, n = 6) were inoculated with 10^4^, 10^5^, 10^6^ or 10^7^ PFU VACV-Elstree in 10 μl PBS by intradermal (ID) tail scarification with a 25–29 G needle^58^ or IN with 10^3^, 10^4^, 10^5^ or 10^6^ TCID_50_ H1N1pdm09 in 50 μl PBS. Clinical signs, weight loss and survival were recorded for 14 days, mice were euthanized 14 dpi or earlier when pre-defined humane endpoint criteria were met (>25% body weight loss).

### Priming, rMVA vaccination and challenge

Mice (6–8 weeks old) were divided into seven groups (n = 24) with four subgroups (n = 6) each (Table 1). Animals were either unprimed (subgroup a) or were primed with 10^8^ PFU wtMVA in 100 μl PBS intramuscularly (IM, two immunizations at week 0 and 4, subgroup b), 10^7^ PFU VACV-Elstree (week 4, subgroup c) or 10^4^ TCID_50_ H1N1pdm09 (week 4, subgroup d). VACV-Elstree and H1N1pdm09 were administered as described above at the optimal priming dose determined in the dose-finding experiments mentioned above. After priming, mice received one or two IM vaccinations at week 8 and/or 12 with 10^8^ PFU of rMVA-NP, rMVA-H1, rMVA-H3 and/or rMVA-H5 in 100 μl PBS. Of note, 10^7^ PFU rMVA-H5 was administered at week 8 to the mice of group 6 (Table 1) to establish if pre-existing immunity affects low dose rMVA-HA vaccination and if a boost with a high dose could overcome potential negative effects. After vaccination, mice vaccinated with rMVA-NP or rMVA-H5 were not challenged and were euthanized one (week 13) or two weeks (week 14) after the second vaccination, respectively (Table 1, group 1–2). The remainder of the animals (Table 1, group 3–7) were challenged IN with 10^3^ TCID_50_ A/Vietnam/1194/04 (H5N1) influenza virus four weeks after the second vaccination (week 16) and monitored twice daily. Mice were euthanized when pre-defined humane endpoint criteria (>25% body weight loss) were reached or at seven days post-challenge (week 17). Blood, spleen and/or lung samples were harvested during necropsy. A single mouse in the wtMVA-primed subgroup of the 2 × rMVA-H5 challenge group (group 6, subgroup b) had to be euthanized due to a wound unrelated to the experiment at week 4. One mouse in the H1N1pmd09-prime group of the 1 × rMVA-H1 and 1 × rMVA-H5 group (group 7, subgroup d) had to be euthanized 10 days after priming because humane endpoint criteria were met. These mice were excluded from further analysis. Animal experiments with groups 3 & 7 and 5 & 6 were performed in parallel but at different stages, groups 1 & 2 and group 4 were performed separately. Analysis of the samples was performed collectively for all groups.

### Virus isolation from lungs

Directly after necropsy, all lungs were snap frozen and stored at −80 °C for processing at a later time point. To perform virus isolations, lungs were thawed, lung weight was recorded and lungs were homogenized with a Polytron homogenizer (Kinematica AG) in MDCK infection medium (without FBS). Quadruplicate ten-fold serial dilutions of these samples in MDCK infection medium supplemented with 0.002% TPCK-Trypsin (Lonza) were used to determine the virus titers on MDCK cells as described previously^57^.

### Stimulation and intracellular cytokine staining of splenocytes

During necropsy spleens were collected in Iscove’s Dulbecco’s Medium (IMDM, Lonza) supplemented with 5% FBS and P/S/G for direct preparation of single cell suspensions using 100 µm strainers (Falcon). Erythrocytes were removed from single cell suspensions by treatment with red blood cell lysis buffer (Roche diagnostics). For intracellular cytokine staining, splenocytes were stimulated with 5 µM synthetic peptide (epitope NP_366–374_: ASNENVEIM [Fig. S2] or ASNEMMETM [Fig. 4]) or 1 µg/250,000 cells recombinant HA protein from H5N1 influenza virus A/Vietnam/1203/04 or A/Indonesia/5/05 (Protein Sciences) in IMDM supplemented with GolgiStop and incubated for 6 h at 37 °C. Mock-treated splenocytes and splenocytes stimulated with 50 ng/ml PMA (Sigma-Aldrich) and 0.5 µg/ml ionomycin (Sigma-Aldrich) served as appropriate negative and positive controls. After stimulation, splenocytes were incubated with fluorochrome-labeled antibodies to CD3e^APC-Cy7^ (BD Pharmingen), CD8b^FITC^ (BD Pharmingen), CD4^PerCP^ (BD Pharmingen) and viable cells were identified with Aqua LIVE/DEAD (Invitrogen). Subsequently, cells were fixed and permeabilized using BD Cytofix/Cytoperm^TM^ Plus (BD Biosciences), and incubated with anti-IFN-γ^PacificBlue^ (Biolegend). Samples were acquired on a FACS Canto II and data was analyzed as described previously^55,59^ using FACS Diva software (BD Biosciences).

### Protein Array (PA) assay

Mouse sera collected at week 8 and 16 (Table 1) were used to determine the presence of antibodies to selected antigens by PA as described previously^60,61^. In short, recombinant HA1 derived from influenza viruses A/California/6/2009 (pH1), A/Wyoming/3/2003 (H3 2003), A/Victoria/361/2011 (H3 2011), A/Hong Kong/156/97 (H5 clade 0), A/Vietnam/1194/04 (H5 clade 1), A/Cambodia/R045050/2007 (H5 clade 1.1), A/Indonesia/5/05 (H5 clade 2.1), A/Turkey/15/2006 (H5 clade 2.2), A/Turkey/Germany-MV/R2472/2014 (H5 clade 2.3.4.4), A/goose/Guiyang/337/2006 (H5 clade 4) and A/chicken/Vietnam/NCVD-016/2008 (H5 clade 7), as well as uninfected and wtMVA infected BHK-21 cells lysed in 1% Triton-X100 (Sigma) in PBS supplemented with mini cOmplete™ EDTA free protease inhibitor tablet (Roche) were printed onto nitrocellulose slides by asciFlexarraver (Scienion). Sera were incubated on the slides in Blotto Blocking Buffer (Thermo Fisher Scientific Inc.) supplemented with 0.1% Surfactant-Amps (Thermo Fisher Scientific Inc.). Subsequently, goat-anti-human IgG labelled with AlexaFluor647 (Jackson ImmunoResearch Laboratories Inc.) was used as conjugate and fluorescent signals were measured using a Powerscanner (Tecan Group Ltd). The titer of each serum sample was defined as the interpolated serum concentration generating the 50% point using a four-parameter logistic nonlinear regression model using R (R Statistical Computing, version 3.1.0. Measured titers were corrected for the positive control included on each slide.

### Detection of MVA- or VACV-specific antibodies by ELISA

For detection of VACV-specific antibodies, HeLa cells were mock-treated or infected with VACV-Elstree at MOI 1 and harvested in 1% Triton-X100 in PBS supplemented with mini cOmplete EDTA free protease inhibitor tablet. Similar procedures were used to obtain BHK-21 cell lysates mock-treated or infected with wtMVA. Sera used for detection of MVA- or VACV-specific antibodies were pre-cleared O/N at 4 °C in a 96-well plate with confluent BHK-21 or HeLa cells, respectively. For ELISA, 96-well plates (Corning Costar) were coated overnight at 4 °C with 10–25 µl cell lysate (mock-treated or infected) per well in 0.05 M Carbonate/Bicarbonate pH 9.6. Plates coated with cell lysate were washed and all plates were blocked for 1 h at room temperature (RT) with blocking buffer consisting of PBS supplemented with 0.05% Tween-20 (PBST, Merck-solutions) and 2% milk powder (w/v, Campina). Subsequently, a 3-log dilution series of serum in blocking buffer was prepared, starting dilution 1:10 or 1:30, and 50 μl was transferred to wells of the antigen-coated plates and incubated for 1–1.5 h at RT. Blocking buffer and VACV-positive serum served as appropriate negative or positive control, respectively. Plates were washed with PBST and incubated for 1 h at RT with HRP-conjugated goat-anti-mouse IgG (DAKO) or goat-anti-human IgG (Southern Biotech). Plates were washed with PBST and incubated for 10 min with 50 µl TMB peroxidase substrate (KPL) after which the reaction was stopped with 0.5 M H_2_SO_4_ (Merck). Absorbance was measured at 450 nm using a Tecan infinite F200. The OD_450_ values at a single dilution in the linear area of the curve were determined and analysed. Due to limited amounts of serum, sera were pooled per subgroup (Table 1, n = 3–6). Sera of VACV-primed animals were tested individually in VACV ELISA as much as possible, although in some groups a few samples had to be pooled (indicated in figure legends). The OD_450_ value obtained with mock-infected BHK-21 or HeLa cell lysate was subtracted from the OD_450_ value obtained with the respective infected cell lysate to determine a net OD_450_ response.

### Hemagglutination inhibition (HI) assay

Sera were treated with a receptor-destroying enzyme (cholera filtrate) overnight at 37 °C, followed by heat-inactivation for 1 h at 56 °C. HI assay was performed in a 2-fold serial dilution in duplicate following a standard protocol using 1% turkey erythrocytes and four HA units of an H5N1 reverse genetics influenza virus with HA (without multibasic cleavage site) and NA gene segments of A/Vietnam/1194/04 and the remaining gene segments of influenza virus A/Puerto Rico/8/34 (6 + 2)^62^.

### Plaque reduction assay

Mouse sera were pooled per subgroup (Table 1, n = 2–5) due to limited serum availability. Sera were heat-inactivated for 30 min at 56 °C. A 2-log dilution series – starting dilution 1:10 – was prepared in CEF culture medium and incubated for 2 h with 200 PFU/well wtMVA (mouse sera) or rMVA-GFP (human sera) in a 1:1 ratio at 37 °C. Human serum with antibodies against MVA was used as a positive control. Subsequently, the serum-virus mixture was incubated for 2 h at 37 °C on a confluent monolayer of CEF cells in 96-wells culture plates. Cells were washed with PBS and incubated for 44–48 h 37 °C. CEF used for serology of mouse samples were fixed with acetone/methanol (Sigma-Aldrich). Plates were blocked with 3% FBS in PBS for 1 h. Subsequently, plaques were stained with rabbit anti-VACV (Lister strain, Acris) followed by goat-anti-rabbit HRP-conjugate (Jackson ImmunoResearch Laboratories Inc.). Samples were developed using True Blue (KPL). The percentage of area covered by stained plaques was measured using a CTL immunospot reader with CTL biospot software. CEF used for serology of human samples were fixed with 2% paraformaldehyde (PFA) for 10 min after which fluorescent plaques were detected using a Typhoon™ FLA9500 (GE Healthcare). Plaques were counted using ImageQuant TL Colony v8.1 software (GE Healthcare). The MVA-neutralization titer was determined as the reciprocal of the highest dilution at which the area covered by plaques was below 50% of the average percentage of the area covered (mouse sera, Fig. S4A) or counted spots (human sera, Fig. S4B) in n = 12 wells without any added serum.

### Statistical analysis

Longitudinal body weight data after H1N1pdm09 priming was analyzed using a repeated measures ANOVA model, with time as within factor. One way ANOVA with multiple comparisons was used to compare the normally distributed (according to the Shapiro-Wilk test) VACV-specific antibody responses after priming (week 8), H5-specific PA antibody responses after one vaccination and the 2003/2011 H3-specific PA antibody responses in mice. A Kruskal-Wallis test was used to compare the not normally distributed HI titers against A/Vietnam/1194/04 after a single vaccination, NP- or H5-specific T cell responses and viral lung titers. HI titers below the detection limit (titer 40) were set to a titer of 20 (the highest possible titer below 40). Statistical differences in the MVA-specific PA response were determined relative to the ‘0 × MVA’ control sample using a Kruskal-Wallis test. Furthermore, the ELISA and neutralization titers in human serum samples were compared using a Wilcoxon matched-pairs signed rank test (MVA sera) or a Mann-Whitney test (VACV sera). Neutralization titers in the plaque reduction assay below the detection limit (10) were set to a titer of 5 (the highest possible titer below 10).

## Electronic supplementary material

Supplemental Figures
