# Supplementary material for: Effects of pre-existing orthopoxvirus-specific immunity on the performance of Modified Vaccinia virus Ankara-based influenza vaccines
Source: Sci Rep. 2018 Apr 24;8:6474. doi: 10.1038/s41598-018-24820-2 (PMC5915537; doi:10.1038/s41598-018-24820-2)
Supplement: Supplementary file 1 — Supplemental Figures [file 41598_2018_24820_MOESM1_ESM.pdf]

Effects of pre-existing orthopoxvirus-specific immunity on the performance of Modified Vaccinia virus Ankara-based influenza vaccines

*Arwen F Altenburg[1], Stella E van Trierum[1], Erwin de Bruin[1], Dennis de Meulder[1], Carolien E van de Sandt[1], Fiona RM van der Klis[2], Ron AM Fouchier[1], Marion PG Koopmans[1], Guus F Rimmelzwaan[1§], Rory D de Vries[1]**

[1] Department of Viroscience, Postgraduate School of Molecular Medicine, Erasmus MC, Rotterdam, the Netherlands

[2] Centre for Infectious Disease Control (Cib), National Institute of Public Health and the Environment (RIVM), Bilthoven, the Netherlands

[§] Current address: Research Center for Emerging Infections and Zoonoses (RIZ), University of Veterinary Medicine, Hannover, Germany

* Corresponding author: Dr. Rory D de Vries, Department of Viroscience, Erasmus MC, PO Box 2040, 3000 CA Rotterdam, the Netherlands, email address: r.d.devries@erasmusmc.nl

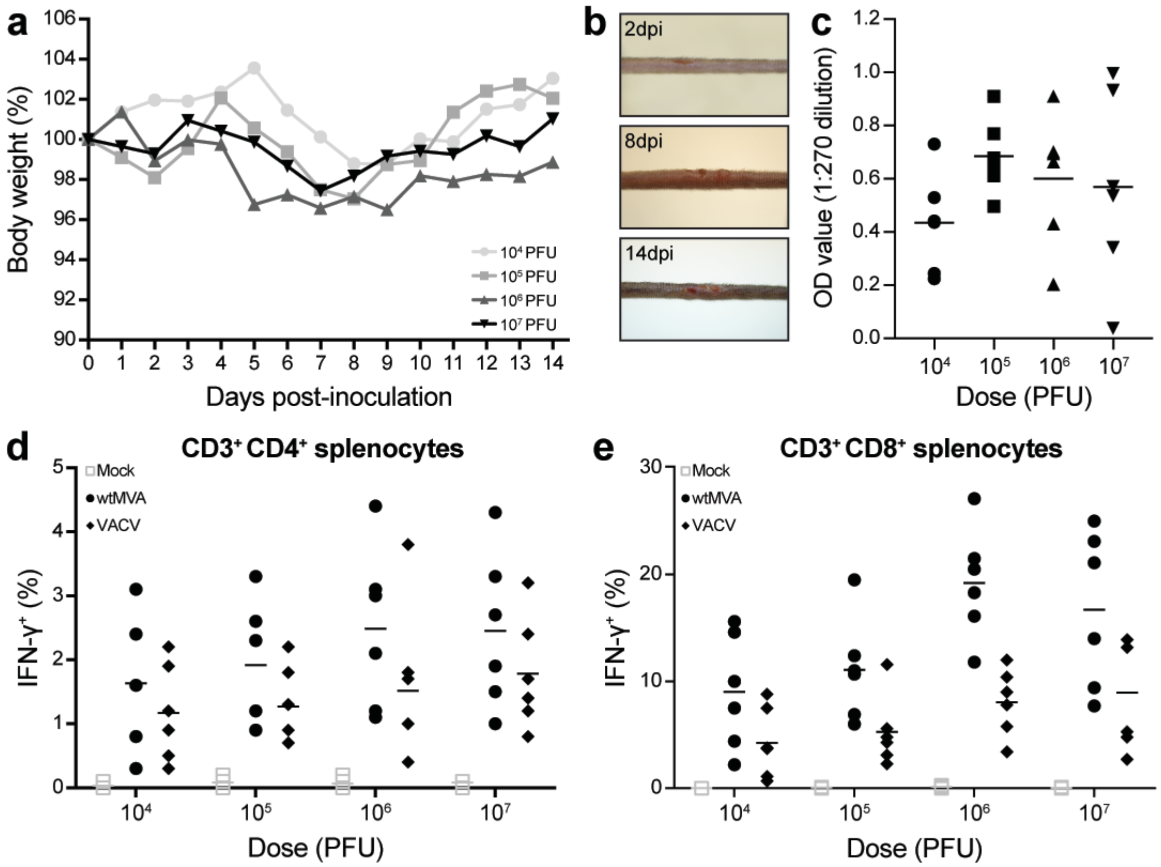

Supplemental Figure 1. VACV-Elstree dose-finding. C57BL/6 mice (n=6 per group) were inoculated with 10^4, 10^5, 10^6 or 10^7 PFU via tail scarification. (**A**) Mean body weight post-inoculation per group. (**B**) Representative images of blister formation at 2, 8 and 14 days post-inoculation (dpi). (**C**) VACV-Elstree specific antibody responses at 14 dpi were measured by ELISA using VACV-infected HeLa cell lysate. The background signal on mock-infected cell lysate was subtracted. The mean is indicated. (**D-E**) Percentage of interferon (IFN)-γ producing $CD3^+CD4^+$ (**D**) and $CD3^+CD8^+$ (**E**) splenocytes after stimulation with wild-type (wt)MVA or VACV at 14 dpi. Unstimulated samples were included as negative control and are shown in grey. The mean is indicated.

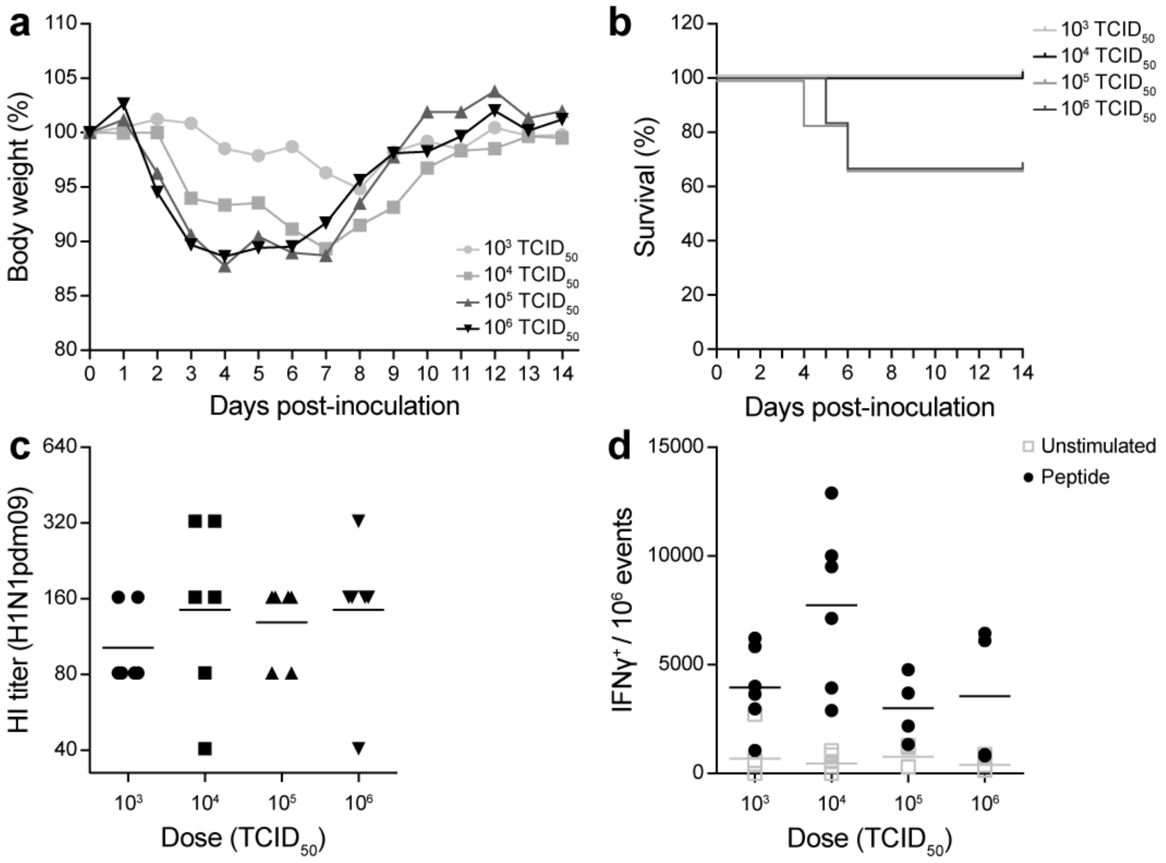

Supplemental Figure 2. H1N1pdm09 dose-finding. C57BL/6 mice (n=6 per group) were inoculated with 10^3, 10^4, 10^5 or 10^6 $TCID_{50}$ influenza virus H1N1pdm09. (**A**) Mean body weight post-inoculation per group. (**B**) Survival curves per group. (**C**) HI antibody titers against H1N1pdm09 of individual mice at 14 dpi. The mean is indicated. (**D**) Number of IFN-γ producing $CD3^+CD8^+$ splenocytes of individual mice after stimulation with $NP_{366-374}$ peptide. Unstimulated samples were included as negative control and are shown in grey. The mean is indicated.

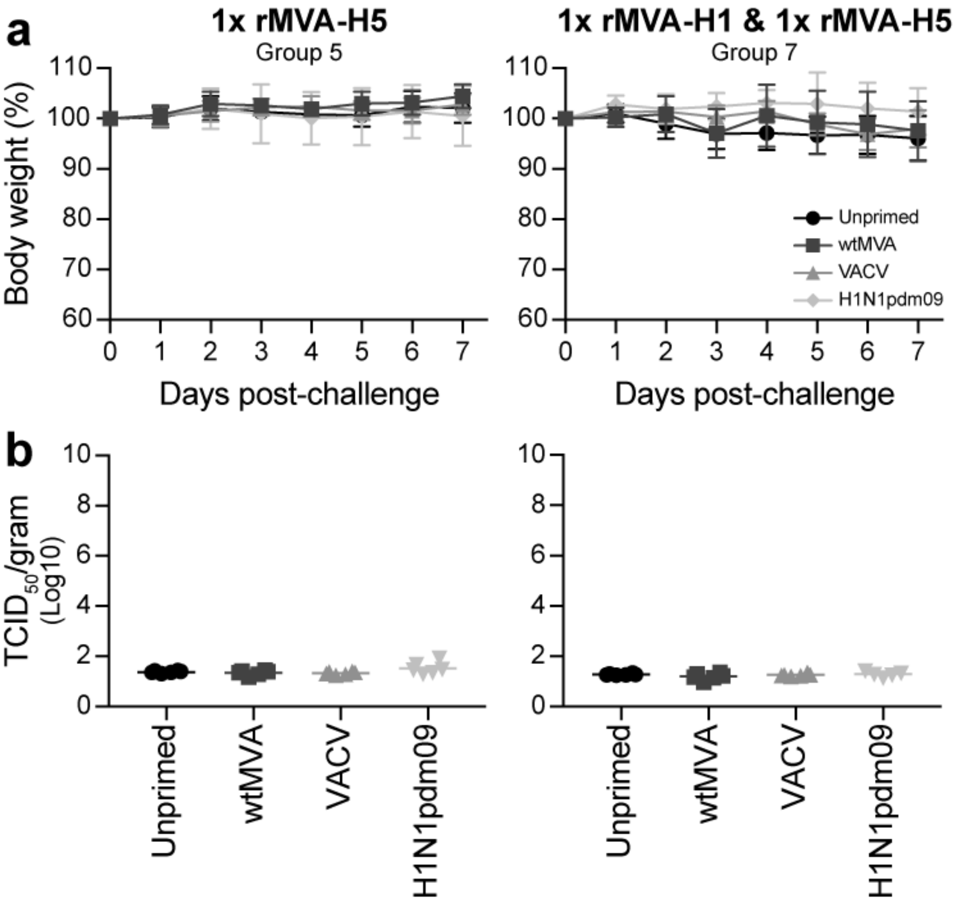

Supplemental Figure 3. Pre-existing immunity does not impair protective capacity of a single rMVA-H5 vaccination. (**A**) Body weight for each of the priming groups after challenge with a lethal dose H5N1 influenza virus, shown for group 5 (one rMVA-H5 vaccination) and group 7 (one rMVA-H1 and one rMVA-H5 vaccination). Mean and standard deviation (SD) are indicated per priming group. (**B**) Viral load in the lungs shown as $TCID_{50}$ per gram lung for each individual animal. Mean is indicated per priming group.

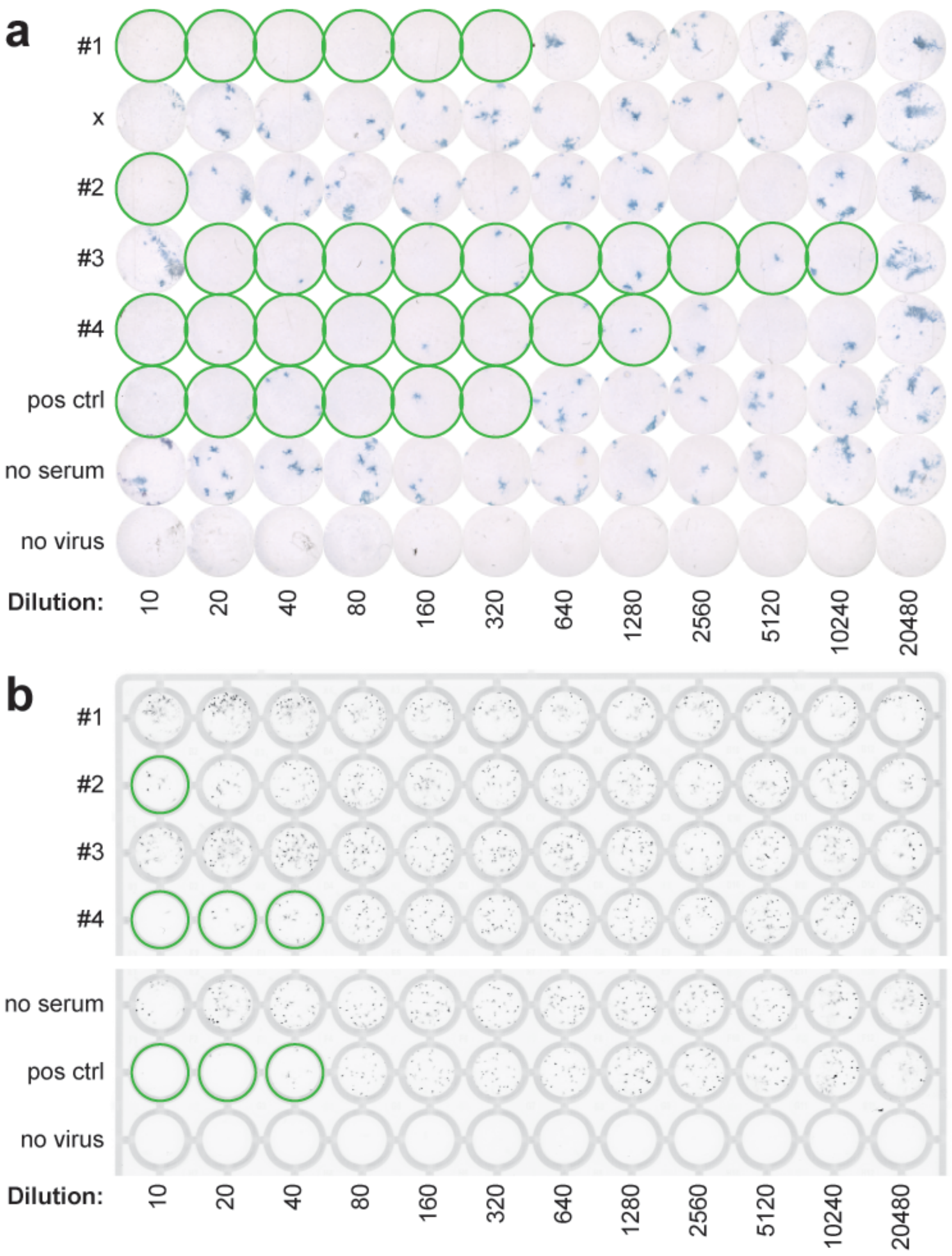

Supplemental Figure 4. Quantification of MVA neutralization in plaque reduction assay. Two-fold serial dilutions of mouse sera (**A**) or human sera (**B**) were incubated with 200 PFU/well wtMVA or rMVA-GFP, respectively. After 2h, the serum-virus mixtures were transferred to CEF cells and incubated for 44-48h. (**A**) For the plaque reduction assay using mouse sera, cells were fixated with acetone and methanol in a 1:1 ratio, followed by staining with rabbit anti-VACV and a goat-anti-rabbit HRP conjugate. Substrate was revealed using True Blue. Shown is a representative image of an CTL immunospot scan. (**B**) For the plaque reduction assay using human sera, cells were fixated with 2% PFA and directly scanned for GFP fluorescence. Shown is a representative image of a Typhoon scan. Neutralization titer was determined as

the reciprocal of the highest dilution at which the area covered by plaques was below background (defined as 50% of the average percentage of the area covered in n=12 wells without any added serum). Wells with values below the cutoff are indicated with a green outline. #1-4 = number of mouse or human samples, x = no serum added.
